# Synthesis of Caffeic Acid Amides Bearing 2,3,4,5-Tetra-hydrobenzo[b][1,4]dioxocine Moieties and Their Biological Evaluation as Antitumor Agents

**DOI:** 10.3390/molecules19067269

**Published:** 2014-06-03

**Authors:** Ji-Wen Yuan, Han-Yue Qiu, Peng-Fei Wang, Jigar A. Makawana, Yong-An Yang, Fei Zhang, Yong Yin, Jie Lin, Zhong-Chang Wang, Hai-Liang Zhu

**Affiliations:** State Key Laboratory of Pharmaceutical Biotechnology, Nanjing University, Nanjing 210093, China

**Keywords:** caffeic acid derivates, EGFR, cell migration inhibition, antitumor activity, structure-activity relationship

## Abstract

A series of caffeic acid amides **D_1_-D_17_** bearing 2,3,4,5-tetrahydrobenzo-[b][1,4]dioxocine units has been synthesized and their biological activities evaluated for potential antiproliferative and EGFR inhibitory activity. Of all the compounds studied, compound **D**_9_ showed the most potent inhibitory activity (IC_50_ = 0.79 *μ*M for HepG2 and IC_50_ = 0.36 μM for EGFR). The structures of compounds were confirmed by ^1^H-NMR, ESI-MS and elemental analysis. Among all, the structure of compound **D_9_** ((E)-N-(4-ethoxyphenyl)-3-(2,3,4,5-tetrahydrobenzo[b][1,4]dioxocin-8-yl)acrylamide) was also determined by single-crystal X-ray diffraction analysis. Compound **D_9_** was found to be a potential antitumor agent according to biological activity, molecular docking, apoptosis assay and inhibition of HepG2.

## 1. Introduction

Cancer is a major cause of death in the World. In the United States one in four deaths is due to cancer [1]. Though we spend a lot of effort and money on research, control of advanced cancer has not been achieved, so it is crucial to find novel cancer agents with new mode of action for saving lives. EGFR (epidermal growth factor receptor) is a growth-factor-receptor tyrosine kinase which plays a vital role in proliferation, survival, migration, differentiation and metastasis of many tumors such as lung-cancer [2,3], head and neck cancer [4,5], breast cancer [6,7], gastric cancer [8], ovarian cancer [9]. In addition, many researchers have demonstrated that the EGFR can be seen as a rational target for anticancer [10,11]. For example, erlotinilb which could inhibit EGFR was approved as an antitumor agent a decade ago [12].

Caffeic acid is a natural phenolic compound found in plants. Caffeic acid and its derivatives possess a wide range of biological activities such as antimicrobial [13], anti-inflammatory [14], antioxidant [15], antimutagenic [16] and anti-HIV [17]. Besides, it also displays potential antitumor activities. Chen *et al.* synthesized caffeic acid phenethyl ester from caffeic acid and found that it could arrest the growth of human leukemia H460 cells [18]. Liao *et al.* have also reported caffeic acid phenethyl ester as a potential antimetastatic agent [19].

Some compounds containing 1,4-benzodioxan units and possessing potent biological activity were reported in previous studies. For example, Lv *et al.* have synthesized a series of luteolin derivatives containing 1,4-benzodioxan which showed better antibacterial activity than luteolin [20]. Sun *et al.* have reported a series of 1,3,4-thiadiazole derivatives containing 1,4-benzodioxan. Among all these compounds, (*E*)-N-(5-(2,3-dihydrobenzo[b][1,4]dioxin-6-yl)-1,3,4-thiadiazol-2-yl)-4-phenylbut-3-enamide showed potent biological activity against HepG2 [21].

However, to our knowledge, few reports were dedicated to synthesizing and evaluating biological activities of caffeic acid derivatives that contain 2,3,4,5-tetrahydrobenzo[b][1,4]dioxocine structures. Thus, herein we described the synthesis and the structure-activity relationship (SAR) of some caffeic acid derivatives with screening for inhibition of cell proliferation activity. In addition, according to EGFR inhibitory activity, molecular docking, apoptosis assay, cytotoxicity and inhibition to cancer cell migration assay, compound **D_9_** was found to be a potential antitumor agent. The results could be helpful to find more potential antitumor agents.

**Scheme 1:**
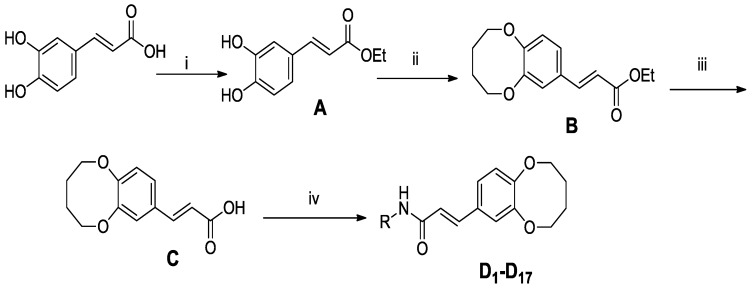
General synthesis of compounds (**D_1_-D_17_**).

*Reagents and conditions*: (**i**) EtOH, HCl, reflux 2 h; (**ii**) 1,4-dibromobutane, DMF, K_2_CO_3_, 70 °C, 6 h; (**iii**) K_2_CO_3_, EtOH-H_2_O, 70 °C, 2 h; (**iv**) R-NH_2_, EDCI, HOBT, CH_2_Cl_2_, 70 °C, 8 h.

## 2. Results and Discussion

### 2.1. Chemistry

**Figure 1:**
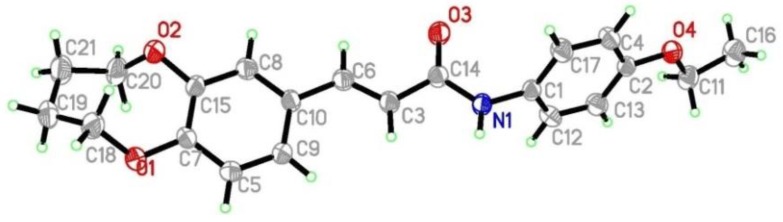
Crystal structure diagram of compound **D_9_**.

In order to study the antitumor activities of (*E*)-3-(2,3,4,5-tetrahydrobenzo[b][1,4]dioxocin-8-yl)acrylic acid amides, compounds **D_1_-D_17_** were synthesized from caffeic acid. The synthetic routes are shown in Scheme 1. All caffeic acid derivatives gave satisfactory analyses. ^1^H-NMR and ESI-MS spectra showed no differences with the designed structures. Besides, the structure of compound **D_9_** was confirmed by X-ray diffraction analysis. The crystal data of **D_9_** is presented in Table 1 and Figure 1, giving perspective views of this compound together with the atomic labeling system.

**Table 1:** Crystal data for compound **D_9_**.

Crystal parameters	Compound D_9_
Empirical formula	C_21_H_23_NO_4_
Molecular weight	353.41
Crystalsize (mm^3^)	0.11 × 0.15 × 0.30
Temperature (K)	273(2)
Radiation	Mo-Kα (0.7103 Å)
Crystalsystem	Monoclinic
Space group	C 2/ *c*
*a* (Å)	26.835(5)
*b* (Å)	9.9597(16)
*c* (Å)	18.698(3)
*α* (°)	90.00
*β* (°)	133.339(4)
*γ* (°)	90.00
*V* (Å^3^)	3635.0(10)
Z	72
*D_c_* (g cm^–^^3^)	1.415
*μ* (mm^–^^1^) absort.coeff	0.127
*F*(000)	1584
*θ* rang (deg)	2.09–25.97
Reflectionscollected	17808(R_int_ = 0.1279)
Indep. reflns	3512
Refns obs. [*I* > 2*σ*(*I*)]	1499
Data/restr./paras	3512/0/236
Goodness-of-fit on *F*^2^	0.984
*R_1_*, *wR_2_* (all data)	0.1310/0.1965
*R_1_*, *wR_2_* [*I > 2σ(I)*]	0.0575/0.0984
Larg. peak/hole (e. Å)	0.181/−0.202

### 2.2. Anti-Proliferative Activity

Compound **C** ((*E*)-3-(2,3,4,5-tetrahydrobenzo[b][1,4]dioxocin-8-yl)acrylic acid), caffeic acid and caffeic acid derivatives **D_1_-D_17_** were evaluated for their anti-proliferation activity against Hela, HepG2 and A431. Erlotinib as standard drug was also tested under the same conditions for comparison. The results were summarized in Table 2.

**Table 2:** Inhibition (IC_50_) of Hela, HepG2 and A431cells proliferation by compounds **D_1_-D_17_** (After cells incubation with compounds for 24 h). 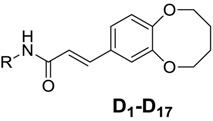

Compounds	R	IC_50_ (μM)		
		Hela	HepG2	A431
**D_1_**	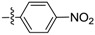	13.64	3.47	16.58
**D_2_**	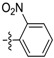	28.17	9.40	24.65
**D_3_**	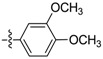	19.62	10.21	8.96
**D_4_**	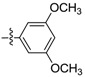	23.72	11.05	10.02
**D_5_**	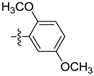	21.40	10.50	9.31
**D_6_**	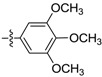	25.68	13.18	11.68
**D_7_**	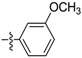	8.71	3.16	12.56
**D_8_**	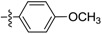	7.04	0.95	7.42
**D_9_**	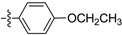	6.75	0.79	7.03
**D_10_**	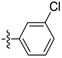	18.54	6.87	20.00
**D_11_**	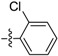	16.70	4.94	18.95
**D_12_**	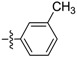	10.77	6.28	14.22	
**D_13_**	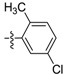	24.30	15.23	14.05
**D_14_**	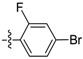	30.75	9.03	22.63
**D_15_**	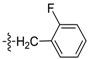	5.15	1.48	17.80
**D_16_**	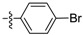	9.04	2.38	7.96
**D_17_**	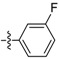	19.52	7.90	21.56
**C**	-	105	56	41
Caffeic acid	-	544	225	93.5
Erlotinib		-	0.08	0.04

As shown in Table 2, caffeic acid derivatives **D_1_-D_17_** showed excellent to moderate activity against HepG2 cell lines displaying IC_50_ values between 0.79 to 15.23 μM. Among them, compound **D_9_** showed the most potent inhibitory activity (IC_50_ = 0.79 µM).

Structure-activity relationships (SAR) of the caffeic derivatives were analyzed. The data of Table 2 showed that compound **C** possessed stronger cell anti-proliferation activity than caffeic acid, suggesting the the 2,3,4,5-tetrahydrobenzo[*b*][1,4]dioxocine structure could enhance the biological activity. That all caffeic amides derivatives showed better cells proliferation activity than compound **C** also indicated that amide groups could enhance the biological activity.

From Table 2, we found that the compounds bearing different amide groups correspondingly possess diverse anti-proliferation activities, which indicated that the anti-proliferative activity of compounds was related to the structure of amides group. For instance, compound **D_1_** bearing one ‑NO_2_ at the *p*-position of the benzene ring showed better anti-proliferation activity as compared to **D_2_** which bears one *m*-NO_2_ on its benzene ring. The same rule also applies to **D_8_** and **D_7_**. The result meant that the *p*-position was important to enhance the anti-proliferative activity of compounds.

Compound **D_3_** bearing -OCH_3_ at the *m*-position possessed lower biological activity as compared to **D_8_** bearing -OCH_3_ at the *p*-position of the benzene ring, making it reasonable to say that the position of the ‑OCH_3_ on the benzene ring also has a distinct impact on anti-proliferation activity. Compound **D_16_** has a -Br at the *p*-position of the benzene ring, and the addition of one -F at the *o*-position led to **D_14_** along with a decrease in the biological activity. From the above fact, one rule could be found: amide groups with one substituent at the *p*-position of the benzene ring tend to display potent cell anti-proliferation activity. Meanwhile, we found that the compounds possessed different biological activities with single diverse *p*-position substituent groups. The inhibitory activity of the derivatives with single different substituents could be arrange in the following order: -OCH_2_CH_3_ > -OCH_3_ > -Br > -NO_2_, indicating that compounds with electron-donating groups at the *p-*position showed better inhibitory activity than those with electron-withdrawing groups.

### 2.3. EGFR Inhibitory Activity

In addition, we selected the top nine compounds having better antiproliferation activity against HepG2 cells to test their EGFR inhibitory activity. The results were summarized in Table 3. Of the compounds studied, most showed good EGFR inhibitory activity. Among all compounds, **D_9_** showed the most potent inhibitory activity with an IC_50_ value of 0.36 μM. The result indicated that **D_9_** has high binding affinity with EGFR which supported the potent anti-proliferation activity.

**Table 3:** EGFR inhibitory activity of synthetic compounds (after enzyme incubation with compounds for 70 min).

Compounds	EGFR (IC_50_ (µM))
**D1**	7.25
**D7**	6.78
**D8**	0.85
**D9**	0.36
**D10**	12.42
**D11**	8.97
**D12**	12.04
**D15**	2.18
**D16**	3.52
Erlotinib	0.03

**Figure 2:**
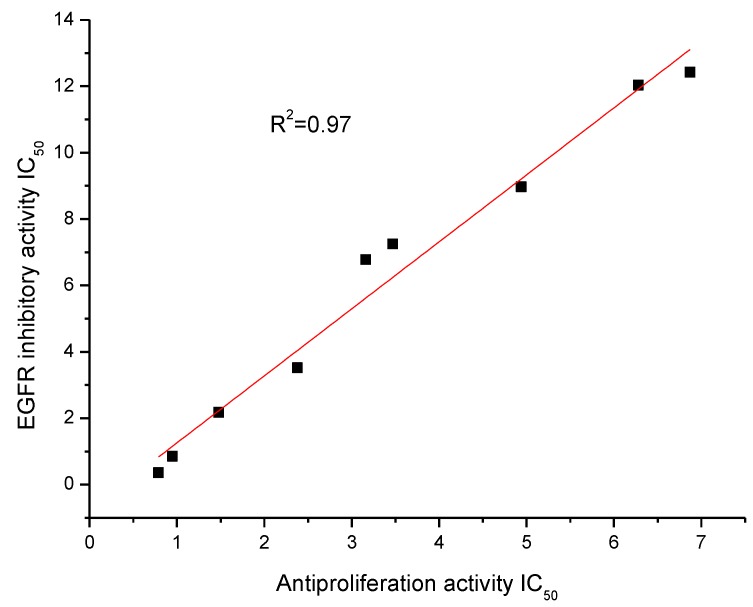
Correlation between the anti-proliferation against HepG2 cell line and EGFR inhibitory activity, R^2^ = 0.97.

An analysis comparing the anti-proliferation activity against the HepG2 cell line and EGFR inhibitory activity of the top nine compounds suggested that there was a moderate correlation between anti-proliferation and EGFR inhibitory (Figure 2, R square value was 0.97). This demonstrated that the potent anti-proliferation activities of the synthesized compounds were probably correlated to their EGFR inhibitory activities.

### 2.4. Apoptosis Assay

**Figure 3:**
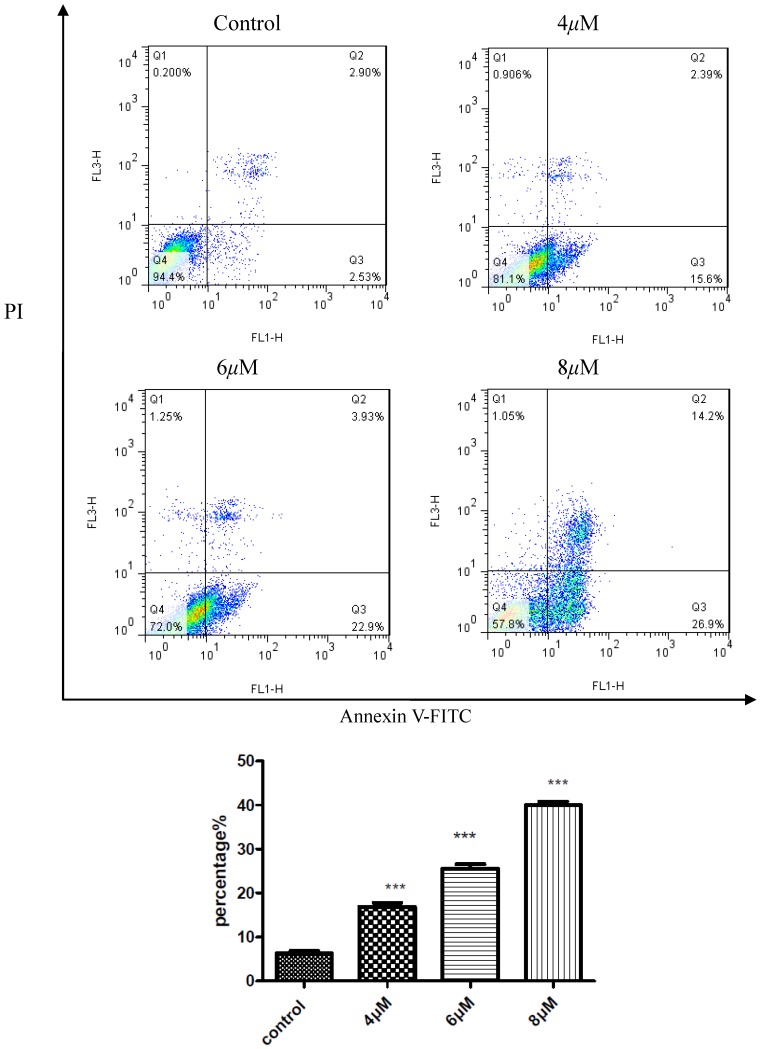
Compound **D_9_** induced apoptosis in HepG2 cell with the density of 4, 6, 8 μM. Data are plotted as the mean ± SD (* *P* < 0.0005 *vs.* control).

To confirm the inhibition of cell growth HepG2, apoptosis study of compound **D_9_** was induced using flow cytometry. As shown in Figure 3, the percentages of cell apoptosis 17.99%, 26.83%, 41.1% were responding to the concentrations of compound **D_9_** 4, 6, 8 μM. The results displayed that compound **D_9_** induced apoptosis of the HepG2 cell line.

### 2.5. Inhibition to HepG2 Cell Migration of D_9_

**Figure 4:**
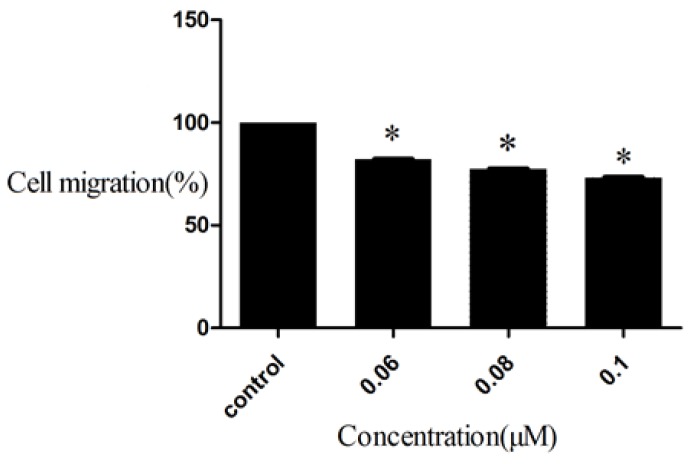
Inhibition to HepG2 Cell migration of compound **D_9._** Values are expressed as a percentage of the control, which was defined as 100%. Data are plotted as the mean ± SD (* *P* < 0.05 *vs.* control).

Though inhibiting cell proliferation could decrease the damage to humans, however many cancer cells can migrate from primary tumors to a distant organ. Metastasis often causes death in patients with cancer. Therefore, the inhibition of cancer cell migration is an effective way for curing cancer. Compound **D_9_** was evaluated for inhibitory ability toward HepG2 cell migration by a Trans well assay. Before testing the inhibition of cell migration, we applied the CCK8 assay to analyze the relationship between concentrations of compound **D_9_** with HepG2 cell survival ratios. The result showed that **D_9_** has no effect to HepG2 cell survival ratios at concentrations lower than 0.1 μM, so we chose **D_9_** concentrations as 0.06, 0.08, 0.1 μM and evaluated their inhibitory activity against HepG2 cell migration. The results were summarized in Figure 4. As shown in Figure 4, the amount of migrating cells was less than control group at a certain concentration of compound **D_9_,** indicating that compound **D_9_** is a potential drug for anti-metastasis therapy.

### 2.6. Molecular Docking

**Figure 5:**
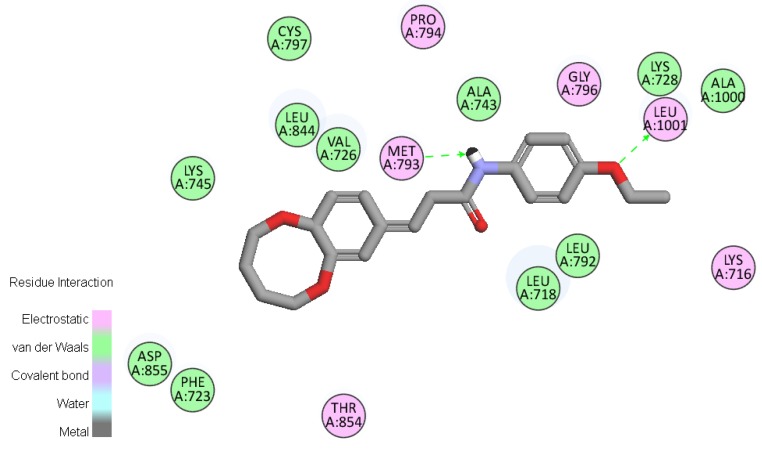
2D molecular docking of compound **D_9_** with 2J6M. The two H-bonds (green lines) are displayed.

**Figure 6:**
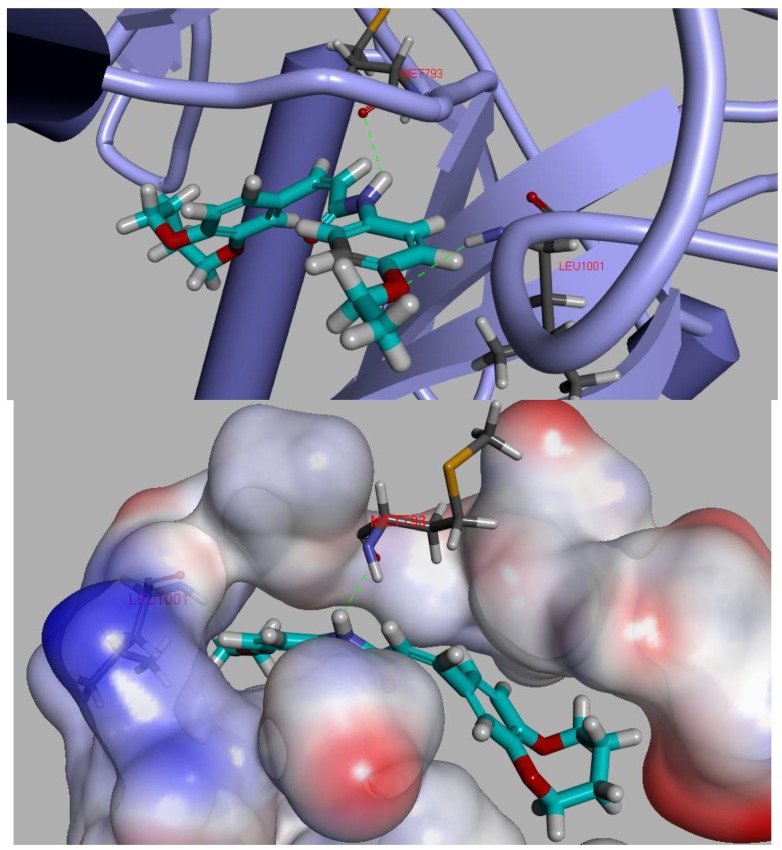
3D model of the interaction between compound **D_9_** and 2J6M bonding site.

To gain better understanding of the interaction binding mode between the target protein and small molecules, the molecular docking of compound **D_9_** and EGFR was performed on the binding model based on the EGFR complex structure (PDB code: **2J6M**) [22] by using Discovery studio 3.5. The 2D and 3D optimal conformation diagrams were depicted as Figure 5 and Figure 6, respectively. In the binding mode, **D_9_** was mixed with amino acid MET 793 (angle N-H-O = 134.33°, distance = 2.11 Å) and LEU1001 (angle O-H-N = 150.6°, distance = 2.44 Å) formed two H-bonds which could enhance the binding affinity resulted in the enhancement in the antitumor activity. The result of molecular docking along with the biological assay data suggested that compound **D_9_** was a potential inhibitor of EGFR.

## 3. Experimental Section

### 3.1. Chemistry

#### 3.1.1. Chemistry General Information

Caffeic acid (>98%, m.p. 222–224 °C) provided by Hubei Yuancheng Pharmaceutical Co., Ltd. was used without purification. The other chemicals and reagents used in our study were made in China with AR grade. ^1^H-NMR spectra were recorded at 300 MHz on a Bruker DPX300 spectrometer (Fällanden, Switzerland), ESI-MS were recorded by a Mariner System 5304 mass spectrometer (Manchester, UK). Elementary analyses were performed on a CHN-O-Rapid instrument (Hanau, Germany). Melting points (uncorrected) were detected on a SPSIC WRS-1B digita1 melting-point apparatus (Shanghai, China). Column chromatography and silica gel (200–300 mesh) were used to separate the compounds. TLC used gel-coated glass slides (silica gel 60 Å GF254) and visualized in UV light (254 nm).

#### 3.1.2. Experimental Procedure for the Synthesis of (*E*)-ethyl 3-(3,4-dihydroxyphenyl)acrylate (**A**)

To a solution of caffeic acid (18 g, 100 mmol) in ethyl alcohol (50 mL), concentrated hydrochloric acid (5 mL) was added. After 2 h of reflux at 70 °C, the solution was evaporated and the solid was washed with water (3 × 100 mL) to give **A** as a yellow power (yield 99%), m.p.: 377–378 °C, ^1^H-NMR (DMSO-*d_6_*, δ ppm): 7.56(d, *J* = 16.2 Hz, 1H, CH); 7.18(d, *J* = 1.5 Hz, 1H, ArH); 7.06 (d, *J* = 1.5 Hz, 1H, ArH); 6.90 (m, 1H, ArH); 6.33 (d, *J* = 15.6 Hz, CH), 4.17 (m, 1H, CH_2_); 1.30 (m, 3H, CH_3_); MS (ESI) 199 [M + H]^+^.Anal. calcd. for C_10_H_14_O_4_: C, 60.59; H, 7.12; O, 32.29 Found: C, 60.62; H, 7.10; O, 32.27.

#### 3.1.3. Experimental Procedure for the Synthesis of (*E*)-ethyl 3-(2,3,4,5-tetrahydrobenzo[b][1,4]-dioxocin-8-yl)acrylate (**B**)

To a solution of **A** (10.4 g, 50 mmol) and potassium carbonate (3.45 g, 25 mmol) in anhydrous DMF (25 mL) at 0 °C, the solution of 1,4-dibromobutane (12 g, 60 mmol) in anhydrous DMF (10 mL) was added dropwise. Then the reaction mixture was heated at 70 °C for 6 h. To the mixture was added water (180 mL) and the solution was extracted by ethyl acetate (3 × 50 mL). The organic solution was evaporated and the residue was purified by a silica gel column eluted with *V*_EtOAc_/*V*_PE_ = 1:10 gave **B** as a yellow solid (39%).

#### 3.1.4. Experimental Procedure for the Synthesis of (*E*)-3-(2,3,4,5-tetrahydrobenzo[b][1,4]dioxocin-8-yl)acrylic acid (**C**)

Compound **B** (6.55 g, 25mmol) and potassium carbonate (3.45 g, 25 mmol) were added to 50% EtOH/H_2_O solution (30 mL) followed by heating at 70 °C for 2 h. Then water (100 mL) was added and the solution was extracted with ethyl acetate. The water layer was decanted and then acidified using dilute hydrochloric acid to pH 1–2 to get **C** as a yellow solid (95%). m.p.: 127–128 °C, ^1^H-NMR (DMSO-*d_6_*, δ ppm): 7.64 (d, *J* = 9.54 Hz, 1H, CH); 7.15 (m, 2H, ArH); 6.97 (d, *J* = 9.51 Hz, 1H, CH); 4.2 (m, 4H, CH_2_); 1.89 (m, 4H, CH_2_); MS (ESI) 235 [M + H]^+^. Anal. calcd. for C_13_H_14_O_4_: C, 66.66; H, 6.02; O, 27.32. Found: C, 66.67; H, 6.05; O, 27.33.

#### 3.1.5. General Experimental Procedure for the Synthesis of Compounds **D_1_-D_17_**

To a mixture of **C** (2.34 g, 10 mmol), HOBT (1.35g, 10 mmol) and EDCI (1.91 g, 10 mmol) dissolved in CH_2_Cl_2_ (30 mL), amine (10 mmol) was added. The solution was refluxed for 8 h. Upon evaporation of CH_2_Cl_2_ and column chromatography (eluent: EtOAc and PE) **D_1_-D_17_** were isolated. 

*(E)-N-(4-nitrophenyl)-3-(2,3,4,5-tetrahydrobenzo[b]*[1,4]*dioxocin-8-yl)acrylamide* (**D_1_**). Yellow solid, yield 31%, m.p.: 125–126 °C, ^1^H-NMR (CDCl_3_, δ ppm): 8.13 (d, *J* = 8.4 Hz, 1H, ArH); 7.97 (d, *J* = 16.1 Hz, CH); 7.51 (m, 3H, ArH); 7.29 (d, *J* = 9.8 Hz, 2H ArH); 7.02 (d, *J* = 8.0 Hz, 1H, ArH); 6.60 (d, *J* = 15.9 Hz, 1H, CH); 4.53–4.57 (m, 2H, CH_2_); 4.31–4.34 (m, 2H, CH_2_); 1.87–1.92 (m, 4H, CH_2_); MS (ESI) 355 [M + H]^+^. Anal. calcd. for C_19_H_18_N_2_O_5_: C, 64.40; H, 5.12; N, 7.91. Found: C, 64.45; H, 5.08; N, 7.98.

*(E)-N-(2-nitrophenyl)-3-(2,3,4,5-tetrahydrobenzo[b]*[1,4]*dioxocin-8-yl)acrylamide* (**D_2_**). Yellow solid yield 32%, m.p.: 119–120 °C, ^1^H-NMR (DMSO-*d_6_*, δ ppm): 9.97 (s, 1H, NH); 8.19 (d, *J* = 8.4 Hz, 1H, ArH); 7.85 (m, 3H, ArH); 7.7 (d, *J* = 8.4 Hz, 1H, ArH); 7.31 (m, 2H, ArH NH); 6.95 (d, *J* = 15.9 Hz, 1H, CH); 6.42 (d, *J* = 15.9 Hz,1H, CH); 4.22–4.41 (m, 4H, CH_2_); 1.87–1.96 (m, 4H,CH_2_); MS (ESI) 355 [M + H]^+^. Anal. calcd. for C_19_H_18_N_2_O_5_: C, 64.40; H, 5.12; N, 7.91. Found: C, 64.43; H, 5.09; N, 7.93.

*(E)-N-(3,4-dimethoxyphenyl)-3-(2,3,4,5-tetrahydrobenzo[b]*[1,4]*dioxocin-8-yl)acrylamide* (**D_3_**). Yellow solid, yield 31%, m.p.: 171–172 °C, ^1^H-NMR (DMSO-*d_6_*, δ ppm): 9.97 (s, 1H, NH); 7.44 (d, *J* = 15.0 Hz, 2H, CH, ArH); 7.2 (m, 3H, ArH); 6.99 (d, *J* = 6.0 Hz, 1H, ArH); 6.91 (d, *J* = 9.0 Hz, 1H, ArH); 6.64 (d, *J* = 18.0 Hz, 1H, CH); 4.25–4.35 (m, 4H, CH_2_); 3.72–3.74 (m, 6H, OCH_3_); 1.92–1.95 (m, 4H, CH_2_); MS (ESI) 370 [M + H]^+^. Anal. calcd. for C_21_H_23_NO_5_: C, 68.28; H, 6.28; N, 3.79. Found: C, 69.01; H, 6.32; N, 3.77.

*(E)-N-(3,5-dimethoxyphenyl)-3-(2,3,4,5–tetrahydrobenzo[b]*[1,4]*dioxocin-8-yl)acrylamide* (**D_4_**). Yellow amorphous solid, yield 34%, m.p.: 115–116 °C, ^1^H-NMR (DMSO-*d_6_*, δ ppm): 10.07 (s, 1H, NH); 7.48–7.43 (d, *J* = 15.72 Hz, 1H, CH); 7.39 (s, 1H); 7.19 (m, 4H, ArH); 6.97 (d, *J* = 8.5 Hz, 1H, ArH); 6.68 (d, *J* = 15.5 Hz_,_ 1H, CH); 4.23–4.34 (m, 4H, CH_2_); 3.37 (s, 3H, OCH_3_); 3.3 (s, 3H, OCH_3_); 1.82 (m, 4H, CH_2_); MS (ESI) 370 [M + H]^+^. Anal. calcd. for C_21_H_23_NO_5_: C, 68.28; H, 6.28; N, 3.79. Found: C, 68.29; H, 6.26; N, 3.81.

*(E)-N-(2,5-dimethoxyphenyl)-3-(2,3,4,5-tetrahydrobenzo[b]*[1,4]*dioxocin-8-yl)acrylamide* (**D_5_**). Yellow amorphous solid, yield 28%, m.p.: 110–111 °C, ^1^H-NMR (DMSO-*d_6_*, δ ppm): 9.19 (s, 1H, NH); 7.93 (s, 1H, ArH); 7.45 (d, *J* = 15.3 Hz, 1H, CH); 7.25 (d, *J* = 8.7 Hz, 2H, ArH); 7.1 (d, *J* = 15.5 Hz, 1H CH); 6.97 (m, 2H, ArH); 6.63 (m, 1H, ArH); 4.2–4.3 (m, 4H, CH_2_); 3.81 (s, 3H, OCH_3_); 3.70 (s, 3H, OCH_3_); 1.84 (m, 4H, CH_2_); MS (ESI) 370 [M + H]^+^. Anal. calcd. for C_21_H_23_NO_5_: C, 68.28; H, 6.28; N, 3.79. Found: C, 68.25; H, 6.29; N, 3.78.

*(E)-3-(2,3,4,5-tetrahydrobenzo[b]*[1,4]*dioxocin-8-yl)-N-(3,4,5-trimethoxyphenyl)acrylamide* (**D**_6_). Yellow solid, yield 30%, m.p.: 145–146 °C, ^1^H-NMR (DMSO-*d_6_*, δ ppm): 9.82 (s, 1H, NH); 8.1 (d, *J* = 15.1 Hz, 2H, CH, ArH); 7.8 (d, *J* = 15.1 Hz, 1H, CH); 7.53 (m, 2H, ArH); 7.10 (d, *J* = 4.9 Hz, 1H, ArH); 6.48 (d, *J* = 9.5 Hz, 1H, ArH) 4.22–4.23 (m, 4H, CH_2_); 4.16 (m, 9H, OCH_3_); 1.92 (m, 4H, CH_2_); MS (ESI) 400 [M + H]^+^. Anal. calcd. for C_22_H_25_NO_6_: C, 66.15; H, 6.31; N, 3.51. Found: C, 66.08; H, 6.28; N, 3.49.

*(E)-N-(3-methoxyphenyl)-3-(2,3,4,5-tetrahydrobenzo[b]*[1,4]*dioxocin-8-yl)acrylamide* (**D_7_**). Yellow amorphous solid, yield 31%, m.p.: 104–105 °C, ^1^H-NMR (DMSO-*d_6_*, δ ppm): 9.93 (s, 1H, NH); 7.45 (d, *J* = 15.7 Hz, 1H, CH); 7.3 (m, 4H, ArH); 7.24 (d, *J* = 7.8 Hz, 2H, ArH); 7.0 (d, *J* = 7.68 Hz, 1H, ArH); 6.65 (d, *J* = 15.9 Hz,1H, CH); 4.24–4.35 (m, 4H, CH_2_); 3.65 (s, 3H, OCH_3_); 1.84 (m, 4H, CH_2_); MS (ESI) 340 [M + H]^+^. Anal. calcd. for C_20_H_21_NO_4_: C, 70.78; H, 6.24; N, 4.13. Found: C, 70.80; H, 6.27; N, 4.16.

*(E)-N-(4-methoxyphenyl)-3-(2,3,4,5-tetrahydrobenzo[b]*[1,4]*dioxocin-8-yl)acrylamide* (**D_8_**). Yellow solid, yield 30%, m.p.: 112–113 °C, ^1^H-NMR (DMSO-*d_6_*, δ ppm): 9.96 (s, 1H, NH); 7.60 (d, *J* = 9.0 Hz, 2H, ArH); 7.44 (d, *J* = 15.0 Hz, 1H, CH); 7.23 (d, *J* = 6.0 Hz, 2H, ArH); 6.99 (d, *J* = 9.0 Hz, 1H, ArH); 6.90 (d, *J* = 6.0 Hz, 2H, ArH); 6.65 (d, *J* = 15.0 Hz, 1H, CH); 4.31–4.25 (m, 4H, CH_2_); 3.73 (s, 3H, OCH_3_); 1.80–1.85 (m, 4H, CH_2_); MS (ESI) 340 [M + H]^+^. Anal. calcd. for C_20_H_21_NO_4_: C, 70.78; H, 6.24; N, 4.13. Found: C, 70.75; H, 6.25; N, 4.17.

*(E)-N-(4-ethoxyphenyl)-3-(2,3,4,5-tetrahydrobenzo[b]*[1,4]*dioxocin-8-yl)acrylamide* (**D_9_**). Yellow solid, yield 30%, m.p. :137–139 °C, ^1^H-NMR (DMSO-*d_6_*, δ ppm): 9.93 (s, 1H, NH); 7.58 (d, *J* = 5.3 Hz, 2H, ArH); 7.44 (d, *J* = 9.4 Hz, 1H, CH); 7.22 (d, *J* = 5.8 Hz, 2H ArH); 6.99 (d, *J* = 4.9 Hz, 1H, ArH); 6.89 (d, *J* = 5.3 Hz, 2H, ArH); 6.66 (d, *J* = 9.33 Hz, 1H, CH); 3.9–4.1 (m, 4H, CH_2_); 3.46 (m, 2H, CH_2_); 1.85 (m, 4H, CH_2_); 1.32 (m, 3H, CH_3_); MS (ESI) 354 [M + H]^+^. Anal. calcd. for C_21_H_23_NO_4_: C, 71.37; H, 6.56; N, 3.96. Found: C, 71.35; H, 6.52; N, 3.97.

*(E)-N-(3-chlorophenyl)-3-(2,3,4,5-tetrahydrobenzo[b]*[1,4]*dioxocin-8-yl)acrylamide* (**D_10_**). Yellow amorphous solid, yield 34%, m.p.: 109–110 °C, ^1^H-NMR (DMSO-*d_6_*, δ ppm): 10.28 (s, 1H, NH); 7.92 (s, 1H, ArH); 7.50 (d, *J* = 2.7 Hz, 1H, ArH); 7.48 (d, *J* = 4.5 Hz, 1H, ArH); 7.35 (d, *J* = 14.7 Hz, 1H, CH); 7.24 (m, 2H, ArH); 7.11 (d, *J* = 4.7 Hz, 1H, ArH); 6.99 (d, *J* = 4.7 Hz, 1H, ArH); 6.64 (d, *J* = 15.0 Hz, 1H, CH); 4.23–4.37 (m, 4H); 1.84–1.95 (m, 4H); MS (ESI) 344 [M + H]^+^. Anal. calcd. for C_19_H_18_ClNO_3_: C, 66.38; H, 5.28; N, 4.07. Found: C, 66.35; H, 5.30; N, 4.07.

*(E)-N-(2-chlorophenyl)-3-(2,3,4,5-tetrahydrobenzo[b]*[1,4]*dioxocin-8-yl)acrylamide* (**D_11_**). Yellow solid, yield 30%, m.p.: 127–128 °C, ^1^H-NMR (DMSO-*d_6_*, δ ppm): 10.26 (s, 1H, NH); 7.98 (d, *J* = 4.9 Hz, 1H, ArH); 7.70 (d, *J* = 4.9 Hz, 1H, ArH); 7.54 (m, 1H, ArH); 7.49 (d, *J* = 9.48 Hz, 1H, CH); 7.42 (m, 1H, ArH); 7.28 (m, 2H, ArH); 6.95 (d, *J* = 4.7 Hz, 1H, CH); 6.38 (d, *J* = 9.72 Hz, 1H, CH); 4.5–4.7 (m, 4H, CH_2_); 1.8–1.9 (m, 4H, CH_2_); MS (ESI) 345 [M + H]^+^. Anal. calcd. for C_19_H_18_ClNO_3_: C, 66.38; H, 5.28; N, 4.07. Found: C, 66.41; H, 5.26; N, 4.12.

*(E)-3-(2,3,4,5-tetrahydrobenzo[b]*[1,4]*dioxocin-8-yl)-N-m-tolylacrylamide* (**D_12_**). Yellow amorphous solid, yield 31%,m.p:112–113 °C, ^1^H-NMR (300 MHz, DMSO-*d_6_*, δ ppm): 10.0 (s, 1H, NH); 7.47 (m, 3H, CH, ArH); 7.21 (m, 3H, ArH); 6.99 (m, 1H, ArH); 6.87 (m, 1H, ArH); 6.64 (d, *J* = 15.66 Hz, 1H, CH); 4.21–4.36 (m, 4H, CH_2_); 2.27 (s, 3H, CH_3_); 1.82–1.84 (m, 4H, CH_2_); MS (ESI) 324 [M + H]^+^. Anal. calcd. for C_20_H_21_NO_3_: C, 74.28; H, 6.55; N, 4.33. Found: C, 74.30; H, 6.56; N, 4.22.

*(E)-N-(5-chloro-2-methylphenyl)-3-(2,3,4,5-tetrahydrobenzo[b]*[1,4]*dioxocin-8-yl)acrylamide* (**D_13_**). Yellow solid, yield 31%, m.p.: 107–108 °C, ^1^H-NMR (DMSO-*d_6_*, δ ppm): 9.55 (s, 1H, NH); 7.84 (d, *J* = 2.1 Hz, 1H, ArH); 7.50 (d, *J* = 15.5 Hz, 1H, CH); 7.24–7.23 (m, 4H, ArH); 7.12 (m, 1H, ArH); 6.65 (d, *J* = 15.7 Hz, 1H, CH); 4.18–4.32 (m, 4H, CH_2_); 2.24 (s, 3H, CH_3_); 1.82–1.86 (m, 4H, CH_2_); MS (ESI) 358 [M + H]^+^. Anal. calcd. for C_20_H_20_ClNO_3_: C, 67.13; H, 5.63; N, 3.91. Found: C, 67.18; H, 5.59; N, 3.87.

*(E)-N-(4-bromo-2-fluorophenyl)-3-(2,3,4,5-tetrahydrobenzo[b]*[1,4]*dioxocin-8-yl)acrylamide* (**D_14_**). Yellow solid, yield 0.32%, m.p.: 110–111 °C, ^1^H-NMR (DMSO-*d_6_*, δ ppm): 9.96 (s, 1H, NH); 7.75 (m, 3H, ArH, CH); 7.53 (m, 2H, ArH); 7.45 (m, 1H, ArH); 7.02 (m, 1H, ArH); 6.37 (d, *J* = 15.9 Hz, 1H, CH); 4.31–4.39 (m, 4H, CH_2_); 1.86–1.92 (m, 4H, CH_2_); MS (ESI) 407 [M + H]^+^. Anal. calcd. for C_19_H_17_BrFNO_3_: C, 56.17; H, 4.22; N, 3.45. Found: C, 56.21; H, 4.20; N, 3.42.

*(E)-N-(2-fluorobenzyl)-3-(2,3,4,5-tetrahydrobenzo[b]*[1,4]*dioxocin-8-yl)acrylamide* (**D_15_**). Yellow solid, yield 30%, m.p.: 86–87 °C, ^1^H-NMR (DMSO-*d_6_*, δ ppm): 8.51 (s, 1H, NH); 7.40–7.46 (m, 1H, ArH); 7.20–7.30 (m, 4H, ArH, CH); 6.97 (d, *J* = 8.7 Hz, 2H, ArH); 6.85 (d, *J* = 8.2 Hz, 1H, ArH); 6.60 (d, *J* = 15.1 Hz, 1H, CH); 4.47 (m, 2H, CH_2_); 4.17–4.43 (m, 4H, CH_2_); 1.79–1.82 (m, 4H, CH_2_); MS (ESI) 342 [M + H]^+^. Anal. calcd. for C_20_H_22_FNO_3_: C, 70.37; H, 5.91; N, 4.10. Found: C, 70.40; H, 5.87; N, 4.11.

*(E)-N-(4-bromophenyl)-3-(2,3,4,5-tetrahydrobenzo[b]*[1,4]*dioxocin-8-yl)acrylamide* (**D_16_**). Yellow amorphous solid, yield 33%, m.p.: 107–108 °C, ^1^H-NMR (DMSO-*d_6_*, δ ppm): 10.2 (s, 1H, NH); 7.66 (d, *J* = 9.0 Hz, 2H, ArH); 7.53 (m, 3H, ArH, CH); 7.24 (d, *J* = 6.0 Hz, 2H, ArH); 6.99 (d, *J* = 9.0 Hz, 1H, ArH); 6.66 (d, *J* = 15.0 Hz, 1H, CH); 4.32–4.37 (m, 4H, CH_2_); 1.82–1.85 (m, 4H, CH_2_); MS (ESI) 389 [M + H]^+^. Anal. calcd. for C_19_H_18_BrNO_3_: C, 58.78; H, 4.67; N, 3.61. Found: C, 58.72; H, 4.69; N, 3.68.

*(E)-N-(3-fluorophenyl)-3-(2,3,4,5-tetrahydrobenzo[b]*[1,4]*dioxocin-8-yl)acrylamide* (**D_17_**). Yellow solid, yield 30%, m.p.: 116–117 °C, ^1^H-NMR (DMSO-*d_6_*, δ ppm): 10.2 (s, 1H, NH); 7.72 (d, *J* = 7.1 Hz, 1H, ArH); 7.49 (d, *J* = 9.3 Hz, 1H, CH); 7.34–7.36 (m, 2H, ArH); 7.23–7.26 (m, 2H, ArH); 7.0 (d, *J* = 4.7 Hz, 1H, ArH); 6.95 (m, 1H, ArH); 6.66 (d, *J* = 9.33 Hz, 1H, CH); 4.23–4.37 (m, 4H, CH_2_); 1.79–1.86 (m, 4H, CH_2_); MS (ESI) 328 [M + H]^+^. Anal. calcd. for C_19_H_18_FNO_3_: C, 69.71; H, 5.54; N, 4.28. Found: C, 69.67; H, 5.56; N, 4.24.

### 3.2. Cell Proliferation Assay

CCK8 is much more convenient and helpful than MTT for analyzing cell proliferation, because it can be reduced to soluble formazan by dehydrogenase in mitochondria and has little toxicity to cells. Cell proliferation was determined using CCK8 dye (Beyotime Inst. Biotech., Nanjing, China) according to the manufacturer's instructions. Briefly, 1–5 × 10^3^ cells per well were seeded on a 96-well plate, and grown at 37 °C for 12 h. Subsequently, cells were treated with all synthesized compounds at increasing concentrations in the presence of 10% FBS for 24 h. After addition of 10 μL CCK8 dyeto each well, cells were incubated at 37 °C for 2 h and plates were read on a Victor-V multilabel counter (Perkin-Elmer, Männedorf, Switzerland) using the default europium detection protocol. Percent inhibition or IC_50_ values of compounds were calculated by comparison with DMSO-treated control wells. The results are shown in Table 2.

### 3.3. Apotosis Assay

Approximately 10^5^ cells/well were plated in a 24 well plate and allowed to adhere. After 24 h, the medium was replaced with fresh culture medium containing compounds **D_9_** at final concentrations of 4, 6, 8 μM. Nontreated wells received an equivalent volume of ethanol (<0.1%). They were trypsinized, washed in PBS and centrifuged at 2,000 rpm for 5 min. The pellet was then resuspended in 500 μL of staining solution (containing 5 μL AnnexinV-FITC and 5 μL PI in Binding Buffer), mixed gently and incubated for 15 min at room temperature (15–25 °C) in dark. The sample were then read in FACScalibur flow cytometer (BD Company, New Jersey, USA) at 488 nm excitation.

### 3.4. General Procedure for Preparation, Purification EGFR, and Inhibitory Assay

A 1.6 kb cDNA encoded for the EGFR cytoplasmic domain (EGFR-CD, amino acids 645–1186) were cloned into baculoviral expression vectors pBlueBacHis2B and pFASTBacHTc, separately. A sequence that encodes (His)6 was located at the 5' upstream to the EGFR sequences. Sf-9 cells were infected for 3 days for protein expression. Sf-9 cell pellets were solubilized at 0 °C in a buffer at pH 7.4 containing 50 mM HEPES, 10 mM NaCl, 1% Triton, 10 μM ammonium molybdate, 100 μM sodium vanadate, 10 μg/mL aprotinin, 10 μg/mL leupeptin, 10 μg/mL pepstatin, and 16 μg/mL benzamidine HCl for 20 min followed by 20 min centrifugation. Crude extract supernatant was passed through an equilibrated Ni-NTA superflow packed column and washed with 10 mM and then 100 mM imidazole to remove nonspecifically bound material. Histidine tagged proteins were eluted with 250 and 500 mM imidazole and dialyzed against 50 mM NaCl, 20 mM HEPES, 10% glycerol, and 1 μg/mL each of aprotinin, leupeptin, and pepstatin for 2 h. The entire purification procedure was performed at 4 °C or on ice.

EGFR kinase assays was set up to assess the level of autophosphorylation based on DELFIA/Time Resolved Fluorometry. Compounds (**D_1_**, **D_7_-D_12_**, **D_15_-D_16_**) were dissolved in 100% DMSO and diluted to the appropriate concentrations with 25 mM HEPES at pH 7.4. In each well, 10 μL compound was incubated with 10 μL (5 ng for EGFR) recombinant enzyme (1:80 dilution in 100 mM HEPES) for 10 min at room temperature. Then, 10 μL of 5× buffer (containing 20 mM HEPES, 2 mM MnCl_2_, 100 μM Na_3_VO_4_, and 1 mM DTT) and 20 μL of 0.1 mM ATP-50 mM MgCl_2_ were added for 1 h. Positive and negative controls were included in each plate by incubation of enzyme with or without ATP-MgCl_2_.

At the end of incubation, liquid was aspirated, and plates were washed three times with wash buffer. A 75 μL (400 ng) sample of europium labeled anti-phosphotyrosine antibody was added to each well for another 1 h of incubation. After washing, enhancement solution was added and the signal was detected by Victor (Wallac Inc., Männedorf, Switzerland) with excitation at 340 nm and emission at 615 nm. The percentage of autophosphorylation inhibition by the compounds was calculated using the following equation:

100% − [(negative control)/(positive control-negative control)]
(1)

The IC_50_ was obtained from curves of percentage inhibition with eight concentrations of compound. As the contaminants in the enzyme preparation are fairly low, the majority of the signal detected by the anti-phosphotyrosine antibody is from EGFR.

### 3.5. Cell Migration Assay

A cell migration assay was conducted as described previously (Qian *et al.* [23]), with a slight modification. In brief, the cells were serum-starved overnight, and the transwells were coated with enhanced chemiluminescence (ECL) cell attachment matrix (Upstate Biotechnology, Lake Placid, NY, USA) at 20 μg/mL. The top chambers of transwells were loaded with 0.2 mL of cells (4 × 10^5^ cells/mL) in serum-free media, and the bottom chambers were loaded with 0.6 mL of MEM media containing 0.5% FBS. The cells were incubated in the transwells at 37 °C in 5% CO_2_ for 24 h. A microplate reader was used to measure the optical density of the eluted solutions in order to determine their migration values. Mean values were obtained from three individual experiments.

### 3.6. Crystal Structure Determination

Crystal structure determination of compound **D_9_** was carried out on a Nonius CAD4 diffractometer equipped with graphite-monochromated Mo-Kα (0.7103 Å) radiation. The structure was solved by direct methods and refined on F2 by full-matrix least squares methods using SHELX-97 [24]. All the hydrogen atoms were placed in calculated position and were assigned fixed isotropic thermal parameters at 1.2 times the equivalent isotropic U of the atoms to which they are attached and allowed to ride on their respective parent atoms. The contributions of these hydrogen atoms were included in the structure-factors calculations. The crystal data, data collection, and refinement parameter for the compound **D_9_** are listed in Table 1. 

CCDC-1005565 contains the supplementary crystallographic data for this paper. These data can be obtained free of charge at www.ccdc.cam.ac.uk/conts/retrieving.html (or from the Cambridge Crystallographic Data Centre (CCDC), 12 Union Road, Cambridge CB2 1EZ, UK; Fax: +44(0)1222-336033; E-Mail: deposit@ccdc.cam.ac.uk).

### 3.7. Molecular Docking Study

Molecular docking of compound **D_9_** into the three dimensional X-ray structure of EGFR (PDB code: 2J6M) was carried out using the Discovery Studio (version 3.5) as implemented through the graphical user interface DS- CDOCKER protocol.

The three-dimensional structures of the aforementioned compounds were constructed using Chem 3D ultra 12.0 software (Cambridge Soft Corporation, Massachusetts, USA), then they were energetically minimized by using MMFF94 with 5000 iterations and minimum RMS gradient of 0.10. The crystal structures of EGFR (PDB code: **2J6M**) complex were retrieved from the RCSB Protein Data Bank. All bound waters and ligands were eliminated from the protein and the polar hydrogen was added to the proteins.

## 4. Conclusions

A series of caffeic acid amides **D_1_-D_17_** bearing 2,3,4,5-tetrahydrobenzo[*b*] [1,4] dioxocin moieties has been synthesized and their biological activities were also evaluated for potential antiproliferative and EGFR inhibitory activity. Among them, the structure of compound **D_9_** was determined by X-ray crystallography. SAR analysis showed that the anti-proliferative activity was affected by 2,3,4,5-tetrahydrobenzo[*b*][1,4] dioxocine structure and *p*-position substituents (-OCH_2_CH_3_ > -OCH_3_ > -Br > -NO_2_) of the benzene ring. Compound **D_9_** displayed the most potent inhibitory activity (IC_50_ = 0.79 μM for HepG2 and IC_50_ = 0.36 μM for EGFR). Docking simulation of compound **D_9_** into the EGFR active site showed that naphthalene ring of **D_9_** with MET 793 formed two H-bonds which enhanced antitumor potency. Therefore, compound **D_9_** may be developed as a potential antitumor agent.

## Supplementary Materials

Supplementary materials can be accessed at: http://www.mdpi.com/1420-3049/19/6/7269/s1.
